# A survey of healthcare providers’ knowledge and attitudes regarding pain relief in labor for women in Ethiopia

**DOI:** 10.1186/s12884-017-1237-4

**Published:** 2017-02-07

**Authors:** Mary McCauley, Catriona Stewart, Birhanu Kebede

**Affiliations:** 1 Centre for Maternal and Newborn Health, Liverpool School of Tropical Medicine, Pembroke Place, Liverpool, L3 5QA UK; 2 Department of Anaesthetics, Yekatit 12 Hospital, Addis Ababa, Ethiopia; 3 Department of Obstetrics, Yekatit 12 Hospital, Addis Ababa, Ethiopia

**Keywords:** Pain relief, Labor, Healthcare providers, Awareness, Ethiopia

## Abstract

**Background:**

To explore healthcare providers’ knowledge and attitudes to the need for pain relief for women in labor.

**Methods:**

A structured questionnaire (*n* = 200) distributed to healthcare providers working in the obstetric departments, including theatres, of three public hospitals in different settings (rural, peri-urban and urban) in Ethiopia. Descriptive analysis was performed using Excel 2013 and SPSS version 22 for associations.

**Results:**

The response rate was 81.5% with 164 questionnaires completed. The majority, 79% of respondents, understood that women can feel moderate to severe pain in labor and 77% were of the opinion that labor pain should be relieved. However, common practices included only supportive measures such as breathing and relaxation exercises, back massage and support from family. The general attitude of healthcare providers is that labor is a natural process, women should be able to cope and that pain relief is not a priority for women in labor. More than half, 52% of healthcare providers had safety concerns with using pharmacological methods to relieve pain in labor.

**Conclusion:**

The majority of healthcare providers understand that women suffer significant pain during labor. However, providing effective pain relief is currently not provided as part of routine intra-partum care in Ethiopia.

**Electronic supplementary material:**

The online version of this article (doi:10.1186/s12884-017-1237-4) contains supplementary material, which is available to authorized users.

## Synopsis

Nearly all healthcare providers understand that women suffer significant pain during labor. However, steps to provide simple medication are not being taken in Ethiopia.

## Background

Childbirth, however fulfilling, can be a very painful experience for women. In many high income countries (HIC), pain relief in labor is considered an essential part of intra-partum care and all women have the choice of and access to a range of pain relief options for labor and delivery [1]. There are many methods to relieve labor pain, both non-pharmacological and pharmacological. The ideal pain relief method must be safe, effective, timely, efficient, equitable, women-centered and ideally should not interfere with labor or the mobility of the laboring women [2]. Non-pharmacological options include the continuous support of a companion, directed breathing and relaxation techniques, massage, laboring in water and the use of transcutaneous electrical nerve stimulation (TENS) in early labor. Pharmacological options include oral tablets (paracetamol, codeine or tramadol), inhalation analgesia (Entonox® - a 50:50 mixture of oxygen and nitrous oxide), intravenous and intramuscular opioids (pethidine or diamorphine) and various types of local (para-cervical or pudendal block) and regional analgesia (epidural or spinal anesthetic).

As the experience of pain in labor is subjective and differs from woman to woman, all woman should have a choice according to her preference and individual circumstances [1]. In low and middle income countries (LMIC), the most common form of pain relief is the continuous support of a companion during labor. The provision of further pain relief in labor is often neglected, against a background of controversy over the need, advantages and disadvantages of pain relief, especially pharmacological options [3]. A lack of awareness, misunderstanding regarding acceptability, safety and availability of pain relief options are considered to be the main reasons why women in many LMIC such as Ethiopia, do not receive adequate pain relief [3, 4].

Ethiopia is a low income country in East Africa with an estimated population of over 9﻿9 million and one of the highest maternal mortality ratios in the world with 353 per 100,000 live births [5]. The percentage of women who attend at least one antenatal visit is 34 and 90% of births are not assisted by skilled healthcare providers [6]. The reasons for women not giving birth in healthcare facilities in Ethiopia are multifactorial and include confidence in traditional birth attendants, misconceptions about services provided at health facilities, inability of family members to be present at time of labor and delivery, traditional and/or spiritual factors, economic factors and accessibility to healthcare facilities [7]. Factors associated with a healthcare facility that influences a woman’s choice of place of delivery in Ethiopia include poor reception on arrival, lack of privacy, shortage of skilled staff and poor quality of care (which may include lack of pain relief options) [7].

Healthcare providers (HCPs) have an important role to play in supporting women’s choice and access to pain relief options during labor. A large systematic review concluded that a woman’s desire for and choice of pain relief during labor is influenced by many factors: personal expectations, support from HCPs, the quality of the relationship between the woman and the HCP and the woman’s involvement in decision making [8].

In many LMIC, HCPs’ awareness of the need for pain relief and the possibilities or choice on how and what to do when, is not well documented. We, therefore, conducted a survey with the aim of exploring HCPs’ awareness of and attitudes to pain relief for women in labor in Ethiopia.

## Methods

We conducted a survey using a paper based structured questionnaire, containing 20 close ended questions. We adapted a questionnaire used in India to assess antenatal women’s awareness and attitudes to pain relief in labor [9]; questions covered socio-demographics including age, sex, occupation, place of work; and other questions assessed knowledge, attitudes and concerns regarding the use of pain relief in labor. Pre-selected options were given and respondents were asked to select all that applied, with the option of ‘other’ in which to provide free text also. We asked HCPs if they were aware of the World Health Organization (WHO) pain ladder in order to gauge an understanding of HCPs towards the ‘step up’ principal of prescribing pain relief in general [10]. The survey was administered to HCPs working in the obstetric department (including obstetric theatres) of three public hospitals providing comprehensive emergency obstetric care in different settings (rural, peri-urban, urban), over a 2-week period in March 2013. Different types of students (nursing, midwifery, medical) were included as HCPs, as in these settings students were working on the wards and were directly involved in service provision for the care of women during and after delivery (under supervision). Descriptive analysis was performed using Excel 2013. Ethical approval was waived from the Research Ethics Committee of Yekatit 12 Hospital as we examined the knowledge and attitudes of HCPs only, using an anonymised questionnaire. This waiver was endorsed by the Head of Department at each hospital, and each HCP was asked for and gave verbal, informed consent prior to the completion of the questionnaire.

## Results

A total of 164 out of 200 HCPs responded (81.5% response rate), 62% of which were female and 38% male with a median age of 24 years. The respondents were drawn from three hospitals: rural (*n* = 32), peri-urban (*n* = 32) and urban (*n* = 100). There was representation from a range of students (nursing, midwifery, medical) and staff (nurses, midwives, pharmacists, anesthetic officers, junior doctors, obstetricians and anesthetists), all HCPs in these settings (Table 1).

**Table 1:** Type of healthcare providers (*n* = 164)

Cadre	%	n
Theatre staff (Anaesthetic nurses, general nurses)	36	59
Labour ward staff (Midwives, obstetric nurses)	22	36
Students (Medical, midwifery, nursing students)	24	39
Doctors (Obstetricians, anaesthetists, junior doctors)	12	20
Other (Pharmacists)	6	10
Total	100	164

### Attitudes regarding pain

**Fig. 1:**
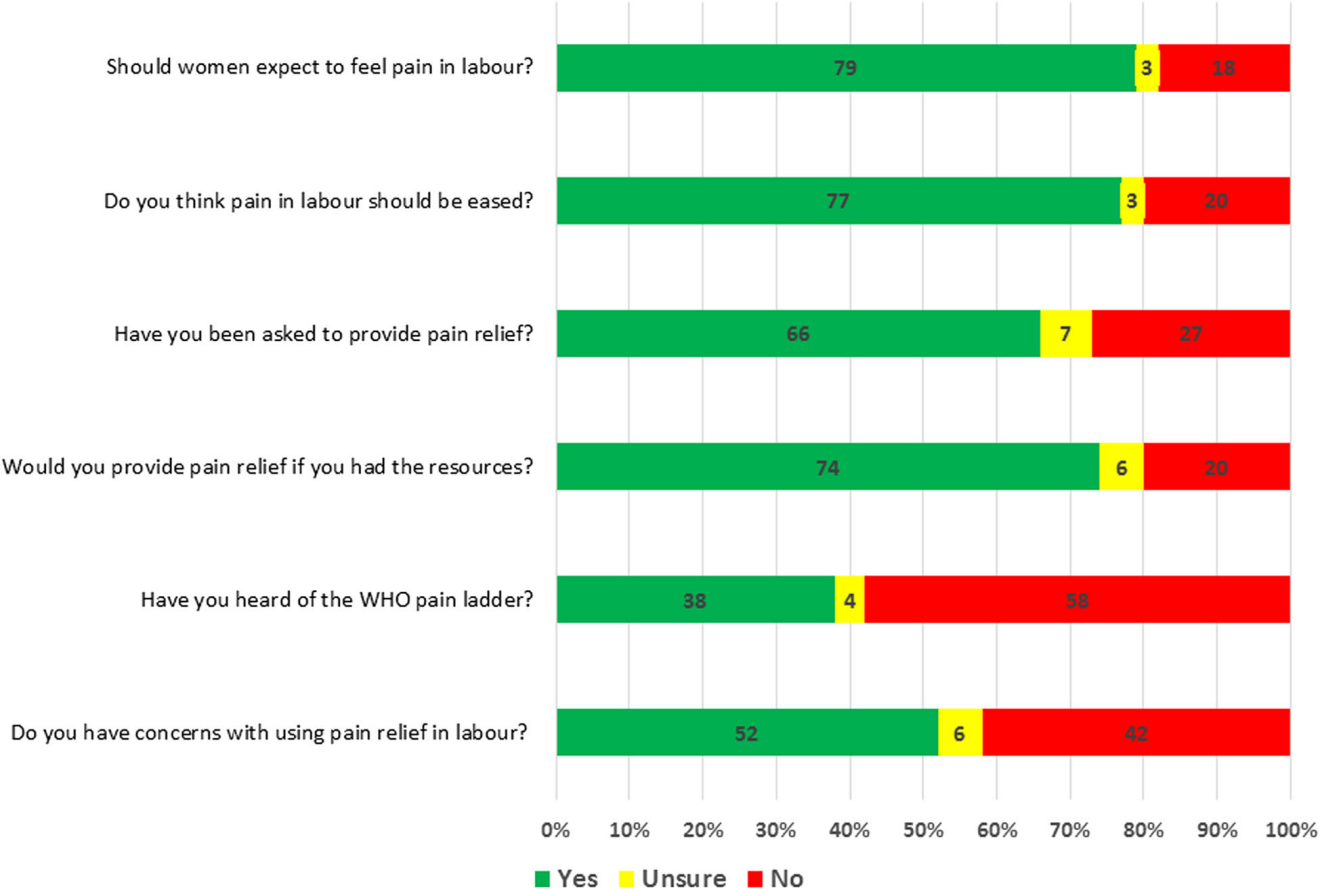
Questions regarding knowledge and attitudes regarding the use of pain relief in labor

The responses to questions regarding knowledge, attitudes and concerns of HCPs regarding the use of pain relief in labor are presented in Fig. 1 and Table 2. The majority (79%) of respondents expected women to feel pain in labor (Fig. 1). Of the 24% (*n* = 30) of respondents who did not think pain should not be relieved reported that: labor is a natural process (38%), pain relief will make the labor longer (19%), pain relief will affect the baby in an adverse way (17%), pain relief may cause complications of labor (20%). Other HCPs routinely recommended a combination of methods of pain relief: personal coping ability, breathing/relaxation exercises, back massage, support from family (Table 2). A total of 98 HCPs (66%) report that they have been asked to provide pain relief for laboring mothers and if they had all of the facilities and resources available, 108 HCPs (74%) reported that they would provide pain relief. Less than half of all HCP (38%) reported knowledge of the WHO pain ladder. In the view of HCPs, the main barriers for patients receiving pain relief included: lack of awareness of HCPs (25%), women’s lack of awareness of pain relief options (18%), pain relief is not a priority for laboring mothers (16%), financial constraints (14%), cultural norms (12%) and lack of availability (4%). A total of 73 HCPs (52%) had concerns about using pain relief in labor. These concerns included fears of adversely affecting the baby, the mother and the delivery process (Table 2).

**Table 2:** Further questions regarding knowledge and attitudes regarding the use of pain relief in labor

Question	Response	%	n
• What level of pain would you expect them to experience? (*n* = 164 answered)	No pain	0	0
	Mild	15	25
	Moderate	33	54
	Severe	52	85
	Total	100	164
• Why do you think pain relief should be relieved? (*n* = 152 answered) (Please tick ALL that apply)	To relieve pain	66	100
	To relieve stress	51	78
	To feel confident	31	47
	To enjoy the experience	34	52
• Why do you think pain relief should not be relieved? (*n* = 40 answered) (Choose MOST relevant)	Labor is a natural process	40	16
	It will make labor longer	20	8
	It will affect the baby	18	7
	Will cause complications	22	9
	Total	100	40
• What types of methods of pain relief do you routinely recommend/practice? (*n* = 137 answered) (Please tick ALL that apply)	None	7	10
	Personal coping ability	57	78
	Breathing exercises	44	60
	Back massage	64	88
	Support from family	68	93
	Warm bath	21	29
	Tablets for pain relief	18	25
	Injectable pain relief	23	31
	Regional analgesia	6	8
• What are the main barriers for patients to receive analgesia in labor? (*n* = 86 answered) (Please tick ALL that apply) • Why do you have concerns with using pain relief?	Lack of awareness of patients	16	14
	Lack of awareness of medical professional	25	21
	Cultural norms	12	10
	Pain relief is not a priority for laboring mothers	18	16
	Financial constraints	12	10
	Availability	3	3
a) It will affect the baby (*n* = 79 answered) (Choose MOST relevant)	It will affect the baby’s breathing	57	45
	It will affect bonding	33	26
	It will affect breast feeding	10	8
	Total	100	79
b) It will affect the labor (*n* = 99 answered) (Choose MOST relevant)	It will stop contractions	23	23
	Make the labor unnatural	17	17
	Affect the mothers ability to push	33	32
	Increase vacuum delivery	11	11
	Increase Caesarean section	13	12
	Total	100	99
c) It will affect the patient (*n* = 42 answered) (Choose MOST relevant)	Physical side effects	71	30
	Patients may feel loss of control	29	12
	Total	100	42

## Discussion

This is the first survey that we are aware of exploring HCPs knowledge and attitudes to pain relief for women in labor in Ethiopia highlighting that there is an attitude that labor is a natural process and that women should be able to cope. We did not include questions to differentiate attitude toward pain relief with regard to the first or second stages of labor, delivery and the postnatal stage or between women undergoing different mode of deliveries e.g. operative vaginal (forceps or vacuum) or abdominal delivery (Caesarean section). In addition, we did not set out (and the sample size was therefore not large enough) to assess if there was an association between age, sex, place of work and cadre of HCP on the awareness and attitudes to the provision of pain relief to women in labor. Going forward, the questionnaire needs to be refined with clearer linkages between questions.

There are a small number of studies conducted in LMIC to assess HCP’s views of pain relief in labor. In our study, the majority of HCPs understood that women can feel moderate to severe pain in labor and most HCPs felt that labor pain should be relieved. This is similar to findings from a study in India among 100 obstetricians where 92% agreed that pain relief in labor was required, with 87% keen for the provision of epidural analgesia [11]. In this study, 45% of obstetricians wanted to provide opioid analgesia for their patients and considered opioids safe, non-invasive, easy to administer and not requiring monitoring or the presence of an anesthetist [11]. The authors conclude that despite a certain degree of awareness there is still a need to further educate, train and to increase communication between obstetricians, anesthetists and women regarding the implementation of effective pain relief in labor in India [11].

There are a number of studies in LMIC to assess women’s views of pain relief in labor. In a study conducted of 124 mothers in Nigeria, only 4% could recall using any form of pain relief in their previous pregnancy [12]. In India, the expectation of labor pain among 205 women was very common although the majority (71%) tolerated it as a natural phenomenon [13]. However, when given the option of pain relief, 98% reported that they would ask for and were prepared to pay for effective pain relief [13]. In South Africa, 56% of 151 pregnant women reported severe pain during a previous labor and 65% of respondents believed that this was unacceptable [14]. Nearly all of the women (99.3%) believed that HCPs had an important role to play in helping to relieve labor pain [14]. In Uganda, 88% of 1293 pregnant women were keen for the pain to be relieved, 79% felt that HCPs should provide this pain relief but only 7% reported knowledge of different options available [15]. For the small number of women who did not want pain relief for their next delivery (10%), their reasons included: wanting a natural childbirth, pain relief was against the will of God, it would harm the baby, they would love their baby more and some women viewed the pain as a form of birth control [15]. There was a similar finding among 450 pregnant women in Niger of which 24% declined pain relief indicating that labor is a ‘natural process’, faith in divine intervention and concerns about side effects [16].

In our study, more than half of all HCPs were concerned about the effect of pain relief on the baby, mother and the labor process. Previous studies have found that these concerns are shared by women as well. One study in Nigeria found that women were afraid of the potential side effects of pain relief on the baby (20%), on themselves (17%) and 3% were concerned regarding the additional cost [12]. However, in South Africa, 78% of pregnant women expressed no concern about potential problems associated with pain relief methods, although 83.4% expressed little confidence in the forms or labor pain relief available in their setting [14].

One large systematic review concluded that a woman’s desire for and choice of pain relief during labor is influenced by many factors: personal expectations, the amount of support from HCPs, the quality of the relationship between the woman and the HCP and the woman’s involvement in decision making [8]. This majority of studies included in this review were conducted in HIC and highlighted that the influences of the attitudes and behaviors of the HCPs can be as important that the influences of pain, pain relief and intra-partum interventions on women’s satisfactions scores of the experience of labor [8].

Communication between a woman and her HCPs is the first step towards preventing distress during labor. The National Institute of Clinical Excellence (NICE) in the UK recommend that all healthcare professionals consider how their own values and beliefs inform their attitude to women coping with pain in labor and that they should ensure their care supports the woman’s choice [1]. In a low resource setting with low levels of utilization of healthcare facilities, such as Ethiopia the provision of pain relief in labor could be a useful incentive to help change negative attitudes and behavior to facility delivery. It is therefore important to include pain relief options as part of any comprehensive, quality of care package offered to women in low resource settings. Whilst the priority in LMIC is to provide evidence-based practices and care (including skilled birth attendance), we argue that equal attention must be given to quality of care, including the provision and experience of care. Quality of care is considered a key component of the right to health and this requires appropriate skills and attitude of HCPs striving to provide a good experience of care for laboring women in a supportive environment [17]. This can be achieved if all HCPs work together in a multidisciplinary approach to refine and implement guidelines for pain relief options for women in labor in LMIC. This should be safe, effective, timely, efficient, equitable, woman-centered and culturally acceptable. Furthermore, efforts must be made to raise the awareness of pain relief options available through education and to empower women and their families to demand pain relief as part of good quality care in labor.

Qualitative research is necessary to explore the underlying reasons for the attitudes of HCPs found in this survey. HCPs working in the community and at primary healthcare facility level also need to be assessed. The timing, best method and benefits of providing cost-effective pain relief in labor needs clarity in a low resource setting.

## Conclusion

This survey demonstrates that HCPs in Ethiopia understand and agree that labor is painful. However, in practice HCPs do not provide women with options for the management of pain during labor other than support from family. There is a need to further educate HCPs to provide pain relief during labor, and for the HCPs to inform women regarding the various types of labor pain relief methods available in order to improve the quality of care given to women in labor.

## Additional file

Additional file 1: Pain Relief Questionnaire Responses. The data comprises the results of the questionnaires administered to 164 healthcare providers in Ethiopia to guage their opinions surrounding administering pain relief to women in labor. (XLSX 17 kb)

## Abbreviations

HCP: Healthcare providers
HIC: High income country
LMIC: Low and middle income countries
NICE: National Institute of Clinical Excellence
TENS: Transcutaneous electrical nerve stimulation
WHO: World Health Organization
